# Supplementary material for: M1BP is an essential transcriptional activator of oxidative metabolism during Drosophila development
Source: Nat Commun. 2023 Jun 2;14:3187. doi: 10.1038/s41467-023-38986-5 (PMC10238438; doi:10.1038/s41467-023-38986-5)
Supplement: Supplementary file 1 — Supplementary Information [file 41467_2023_38986_MOESM1_ESM.pdf]

Supplementary Information

Supplementary Figures

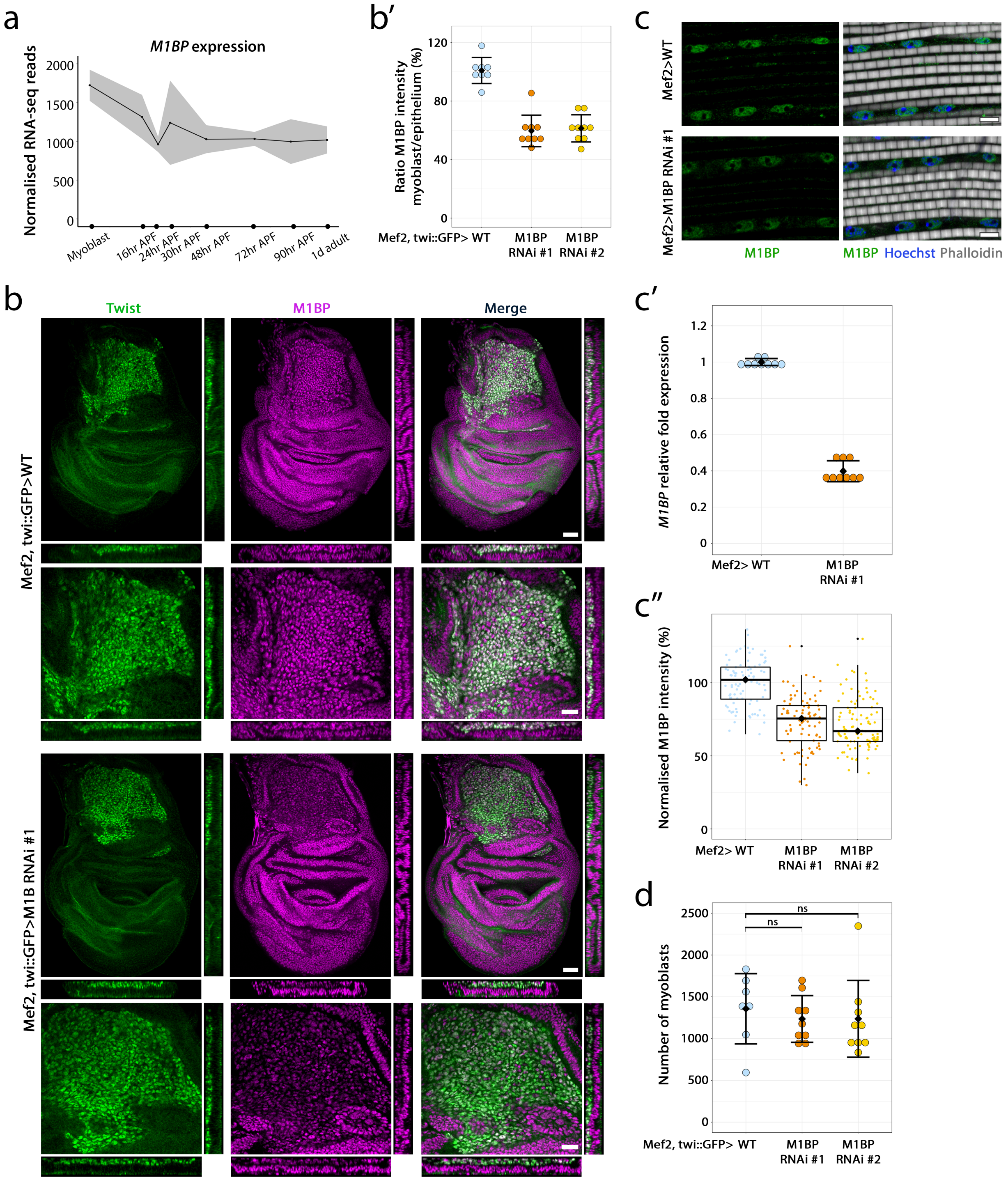

Supplementary Figure 1. M1BP-RNAi efficiently decreases M1BP protein levels during myogenesis

(a) *M1BP* mRNA expression levels during myogenesis. Mean and standard deviation are represented, data analysed from[7]. **(b)** Confocal sections of 0 h APF wild-type (Top) and *M1BP*-depleted (Bottom) wing discs stained with anti-M1BP antibody and anti-GFP to visualise myoblasts, labelled with a twist-GFP transgene. For each section, an enlarged view of the myoblast (GFP-positive) area and orthogonal views are represented. Scale bars correspond to 50 µm for large views and 20 µm for zoomed, bottom views. **(b')** (Top) Quantification of M1BP intensity represented as a ratio of M1BP intensity in the myoblast area

compared to the epithelial area. The mean and standard deviation are shown. Each dot represents a value from one disc (n_{WT} = 9, $n_{\#1}$ = 8 and $n_{\#2}$ = 9 discs from at least 5 animals). **(c)** Confocal sections of WT (left) and M1BP-depleted (right) female adult DLM stained with an anti-M1BP antibody, phalloidin to visualise actin and Hoechst to stain nuclei. Scale bar represents 5µm. **(c')** Relative fold expression of *M1BP* mRNA levels assessed by quantitative RT-PCR in the WT and *M1BP* RNAi condition driven by *Mef-GAL4*. Mean and standard deviation are represented where each point represents a value from a single technical replicate (*n* = 9 replicates for both genotypes over three independent biological replicates). **(c'')** The intensity of M1BP staining is represented in boxplots (median and quartiles) with whiskers (minimum to maximum), where each point represents a value of a single nucleus (n_{WT} = 93, $n_{\#1}$ = 89 and $n_{\#2}$ = 101 nuclei from at least 3 animals). **(d)** Quantification of the number of myoblasts. The mean and standard deviation are shown. Each dot represents a value from one disc (n_{WT} = 7, $n_{\#1}$ = 9 and $n_{\#2}$ = 9 discs from at least 5 animals). After applying Shapiro-Wilk test for normality an unpaired, two-sided Student's-test was applied (p-values = 0.52 and 0.6). Source data are provided as a Source Data File.

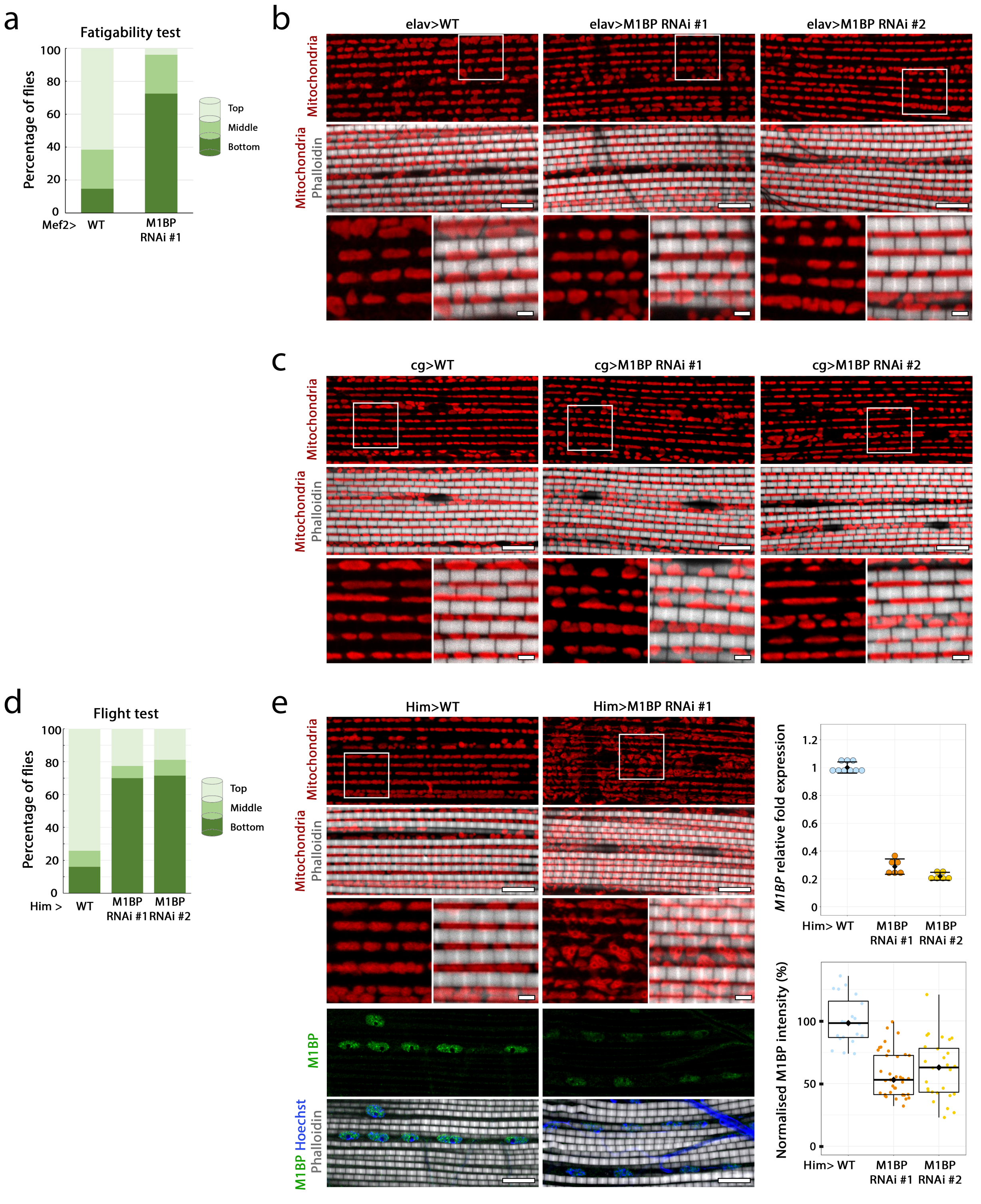

Supplementary Figure 2. Mitochondrial phenotypes observed upon M1BP KD are specific to its expression in the flight muscle.

(a) Flight muscle fatigability was scored as a percentage of male flies (Mef2>WT and Mef2>*M1BP-RNAi* #1) landing on each segment of the column. Represented is the mean of two independent biological replicates (n_{WT}= 204, $n_{\#1}$ = 135 flies). **(b)** Confocal sections of 2-days-old female adult wild-type (left) and *M1BP*-depleted (right) DLM mitochondria, driven by a neuron-specific *elav-GAL4* driver. Throughout the whole figure, for each section, an enlarged view of the individual mitochondrion (depicted by a white rectangle) is

shown. Myofibrils are stained with phalloidin (grey) and mitochondria with an anti-ATP5A antibody (red). Scale bars represent 10 µm for top images and 2 µm for zoomed, bottom views. **(c)** Identical staining and scale bars as **(b)**, with *M1BP-RNAi* being driven by a fat body-specific, *cg-GAL4* driver. **(d)** Flight ability was scored as a percentage of male flies landing on each segment of the column. Tested flies are Him>WT and Him>*M1BP* RNAi. Represented is the mean of three independent biological replicates (n_{WT} = 180, $n_{\#1}$ = 240 and $n_{\#2}$ = 210 flies). **(e)** (Left) Confocal sections of WT (left) and *M1BP*-depleted (right) female adult DLM, driven by *Him-GAL4*. Myofibrils were stained with phalloidin (grey) and mitochondria with an anti-ATP5A antibody (red). Scale bar is 10 µm for top and 2 µm for zoomed, bottom views. M1BP expression in adult female DLM was visualised with anti-M1BP antibody (green), nuclei stained with Hoechst (blue) and myofibrils with phalloidin (grey). Scale bar is 10 µm. (Right) Relative fold expression of *M1BP* mRNA levels assessed by quantitative RT-PCR in the WT and *M1BP* RNAi condition driven by *Him-GAL4*. Mean and standard deviation are represented where each point represents a value from a single technical replicate (*n* (WT) = 9 and *n* (both RNAi) = 6 technical replicates over two independent biological replicates). The intensity of anti-M1BP immunostaining is represented in boxplots (median and quartiles) with whiskers (minimum to maximum), where each point represents a value of a single nucleus, *n*= 5 animals per genotype. Source data are provided as a Source Data File.

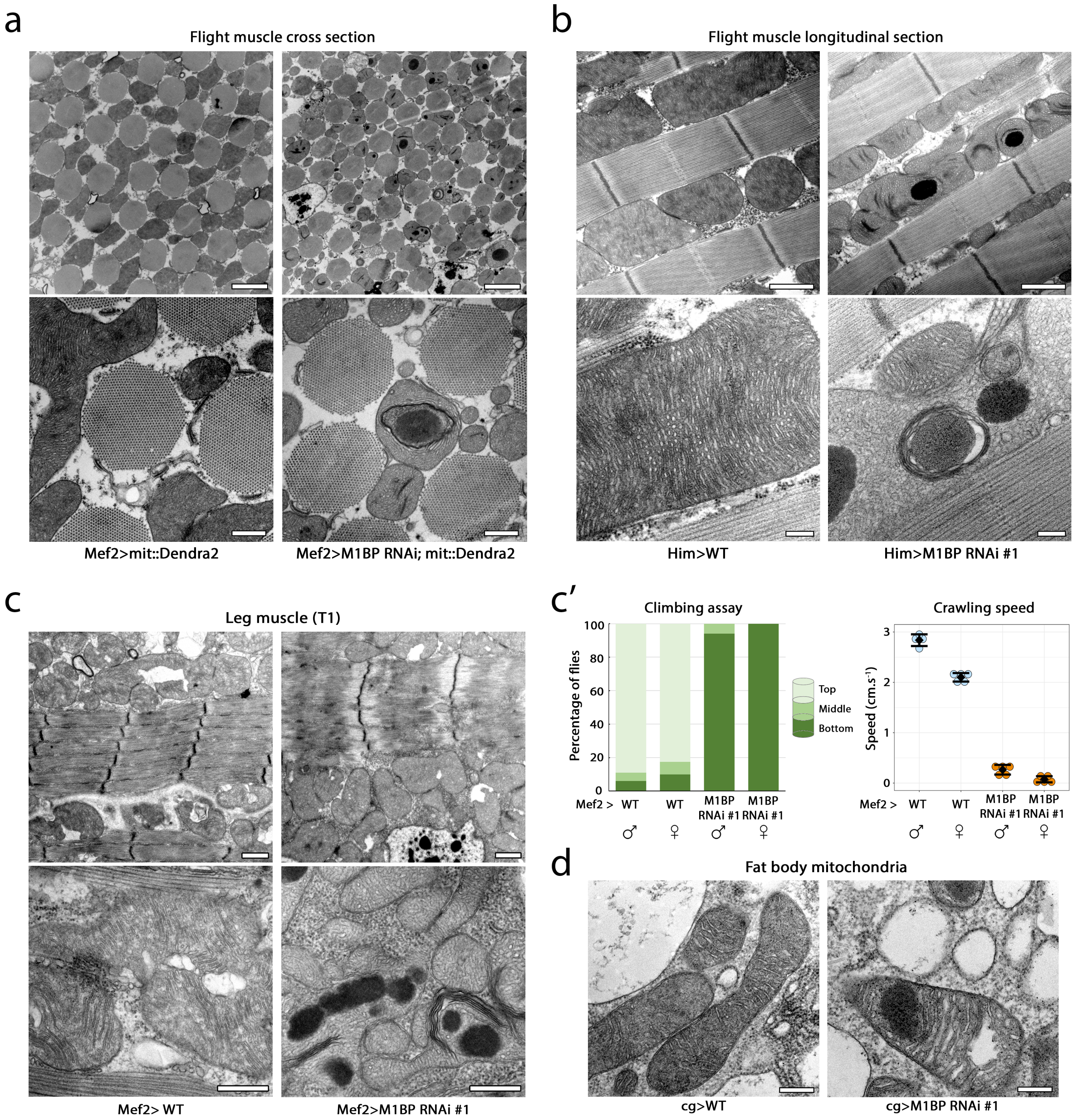

Supplementary Figure 3. M1BP inhibition leads to ultrastructural defects in muscle and fat body mitochondria

(a) Transmission electron microscopy micrographs of male adult WT (left) and *M1BP*-depleted (right) DLM mitochondria using *Mef2-GAL4* driver, cut in cross sections. Scale bars represent 2 µm for top images and 500 nm for larger bottom views. **(b)** Transmission electron microscopy micrographs of male adult WT (left) and *M1BP*-depleted (right) DLM mitochondria driven by *Him-GAL4*. Scale bars represent 1 µm for top images and 200 nm for larger bottom views. **(c)** Transmission electron microscopy micrographs of male adult WT (left) and *M1BP*-depleted (right) mitochondria in T1 adult coxa leg muscles, driven by *Mef*2-*GAL4*. Scale bars represent 1 µm for top images and 500 nm for larger bottom views. **(c’)** Negative geotaxis climbing ability was scored as the mean percentage of flies present in each section of the column (n = 50 flies for each condition examined from five biological replicates). Crawling speed was quantified as the distance travelled during a fixed time, where each dot corresponds to a mean speed value of a biological

replicate (*n* = 5 flies for each condition examined from five biological replicates). Error bars represent standard deviation of the mean. **(d)** Transmission electron microscopy micrographs of 3rd instar larval fat body mitochondria in WT (top) or *M1BP* KD (bottom) condition, driven by *cg-GAL4*. Scale bar is 250 nm. Source data are provided as a Source Data File.

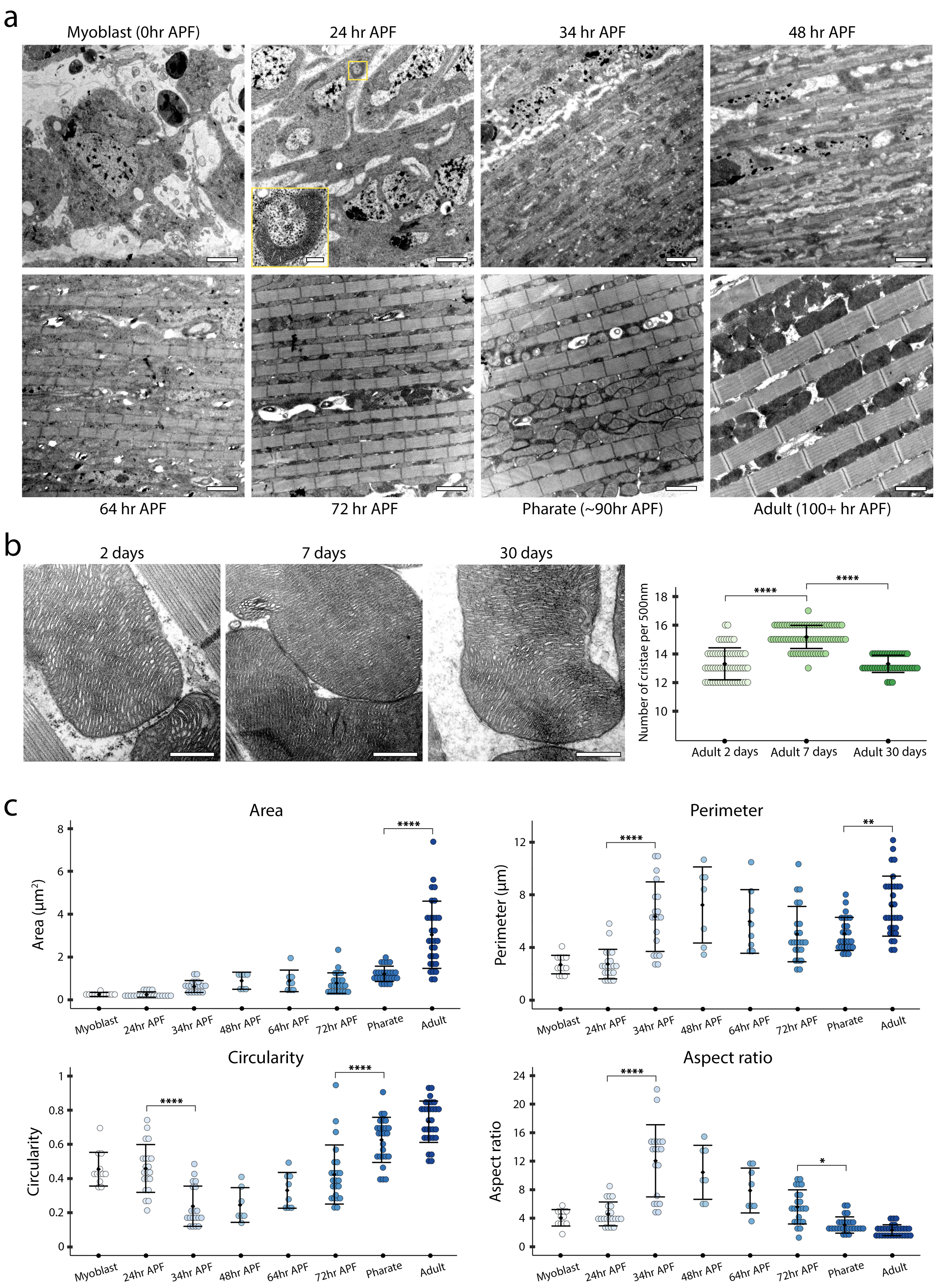

Supplementary Figure 4. Large mitochondrial cristae biogenesis occurs during flight muscle development

(a) Transmission electron microscopy micrographs of wild-type flight muscle development. Scale bars correspond to 2 µm. For 24 h APF a zoomed view of a single mitochondrion, depicted by a yellow rectangle is shown (scale bar is 200 nm). **(b)** Transmission electron microscopy micrographs of wild-type flight

muscles at 2, 7 and 30 days of adult development. Scale bar is 500 nm. The quantification of the number of mitochondrial cristae per length unit (500 nm) is shown. Mean and standard deviation are shown with each point representing a value of a single mitochondrion ($n_{2\ days}$ = 44, $n_{7\ days}$ = 46 and $n_{30\ days}$ = 28 mitochondria). For statistical analysis, one-way analysis of variance (ANOVA) was performed, followed by Tukey's test for multiple mean comparisons, p-values (2 days vs 7 days = 0 and 7 days vs 30 days = 0). **(c)** Quantification of mitochondrial parameters during flight muscle development. Mean and standard deviation are shown with each point representing a value of a single mitochondrion (for all parameters *n* (Myo) = 12, *n* (24 h) = 21, *n* (34 h) = 19, *n* (48 h) = 7, *n* (64 h) = 8, *n* (72 h) = 23, *n* (pharate) = 26 and *n* (adult) = 30 mitochondria). For statistical analysis, one-way analysis of variance (ANOVA) was performed, followed by Tukey's test for multiple mean comparisons. For area p-values (Myo vs 24 h = 1, 24 h vs 34 h = 0.8, 34 h vs 48 h = 1, 48 h vs 64 h = 1, 64 h vs 72 h = 1, 72 h vs pharate = 0.53 and pharate vs adult = 0). For perimeter p-values (Myo vs 24 h = 1, 24 h vs 34 h = 1.3*10^-6, 34 h vs 48 h = 0.97, 48 h vs 64 h = 0.92, 64 h vs 72 h = 0.93, 72 h vs pharate = 1 and pharate vs adult = 0.0026). For circularity p-values (Myo vs 24 h = 1, 24 h vs 34 h = 1.3*10^-5, 34 h vs 48 h = 1, 48 h vs 64 h = 0.91, 64 h vs 72 h = 0.68, 72 h vs pharate = 0 and pharate vs adult = 0.06). For aspect ratio p-values (Myo vs 24 h = 1, 24 h vs 34 h = 0, 34 h vs 48 h = 0.83, 48 h vs 64 h = 0.5, 64 h vs 72 h = 0.34, 72 h vs pharate = 0.01 and pharate vs adult = 0.96). Source data are provided as a Source Data File.

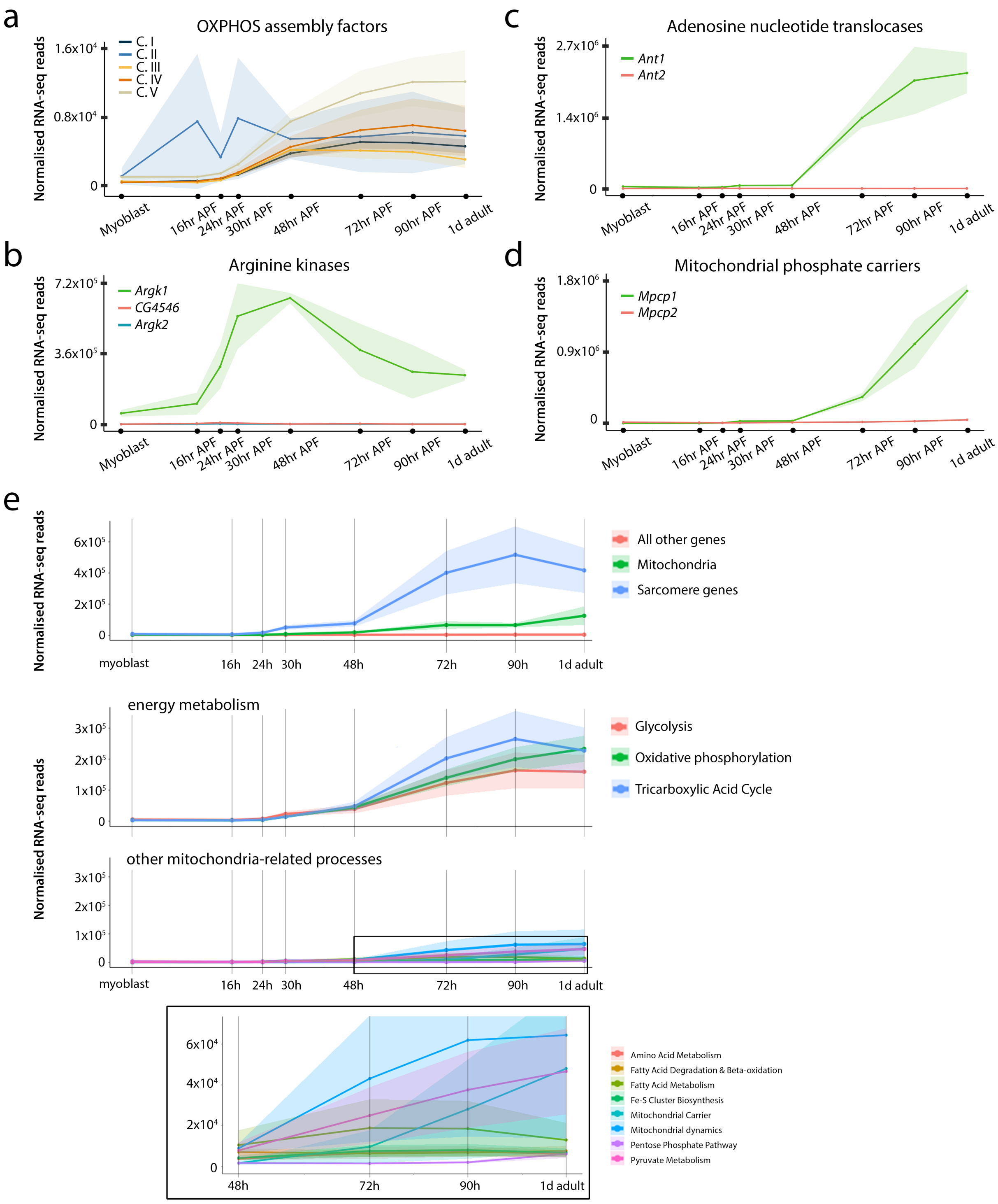

Supplementary Figure 5. Large-scale mitochondrial and sarcomeric gene transcriptional upregulation occurs during flight muscle development

(a) Transcriptional profile during flight muscle development of genes encoding assembly factors of the mitochondrial respiratory chain complexes. These data are the same as those presented in Figure 2c but with the addition of Complex II assembly factor Sirup that was omitted from the main data due to its oscillatory expression during early stages of pupation, which is likely a sequencing artefact. The solid line represents the normalised mean of sequenced counts of all assembly factors of a given respiratory complex

with shaded areas representing the standard error of the mean. **(b, c, d)** The transcriptional profile during flight muscle development of genes encoding arginine kinases **(b)** adenosine nucleotide translocases **(c)**, and mitochondrial phosphate carriers **(d)**. In all cases the solid lines represent the normalised mean of sequenced counts of a given gene with shaded areas representing the standard deviation of the mean. **(e)** Transcriptional profile of sarcomeric, mitochondrial and all other genes during IFM development. For mitochondrial genes, the transcriptional profile of genes related to energy metabolism and other mitochondrial processes (with a zoomed view in the bottom) are represented. In all cases the solid lines represent the normalised mean of sequenced counts of a given gene with shaded areas representing the standard error of the mean. Source data are provided as a Source Data File.

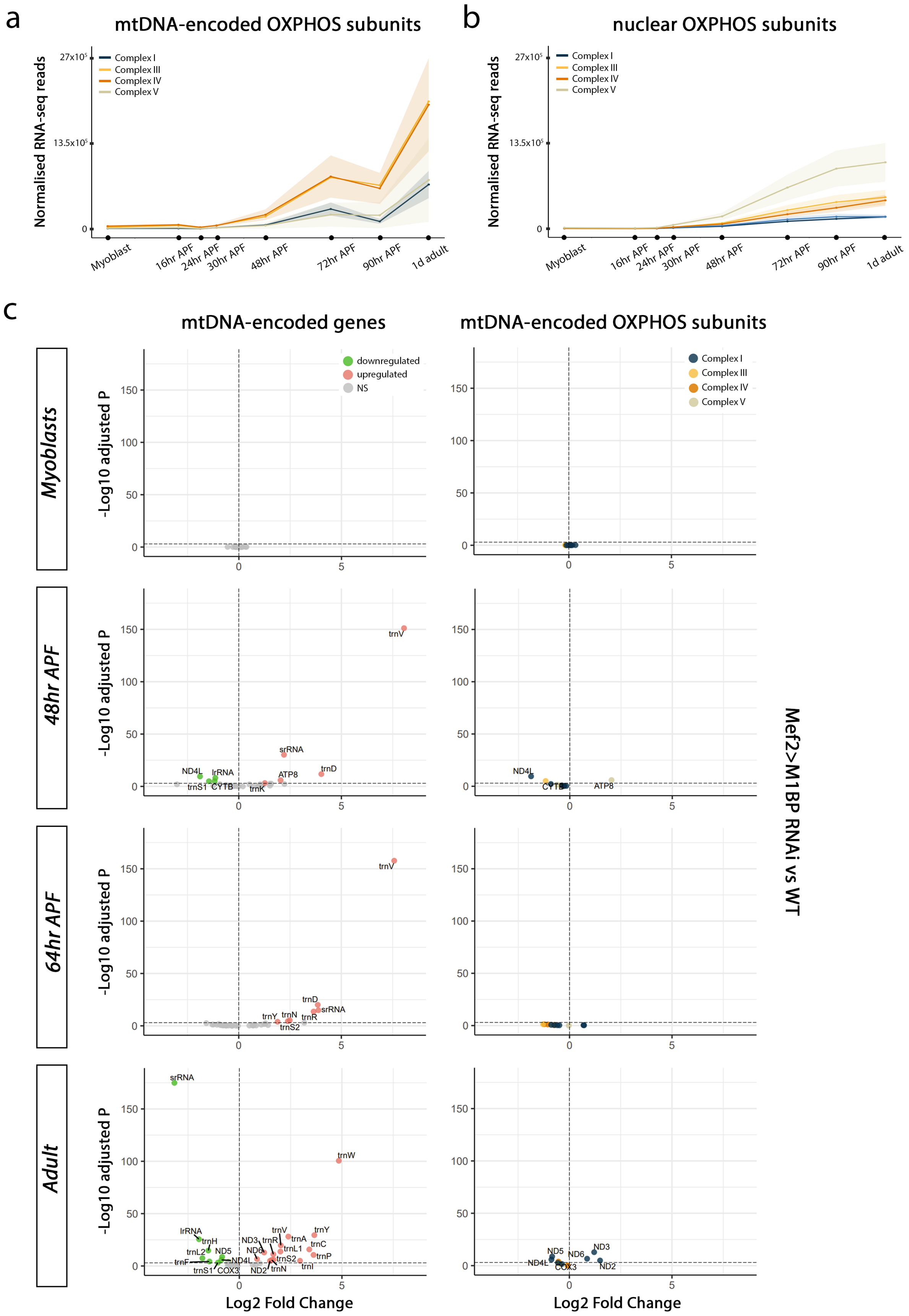

Supplementary Figure 6. M1BP does not transcriptionally regulate mtDNA-encoded OXPHOS subunits

(a,b) Transcriptional profiles using the same ordinate scale during flight muscle development of genes encoded by mtDNA **(a)** and nuclear DNA **(b)** that constitute the mitochondrial respiratory chain complexes.

The solid line represents the normalised mean sequenced counts of all subunits of a given respiratory complex with the shaded areas representing the standard error of the mean. **(c)** Volcano plots of all differentially expressed mtDNA-encoded genes (left) or only mtDNA-encoded, OXPHOS-related (right) genes between Mef2>WT and Mef>*M1BP* RNAi at time points corresponding to analyses in Figure 3. For statistical analysis, the Wald test with Benjamini-Hochberg-correction was applied.

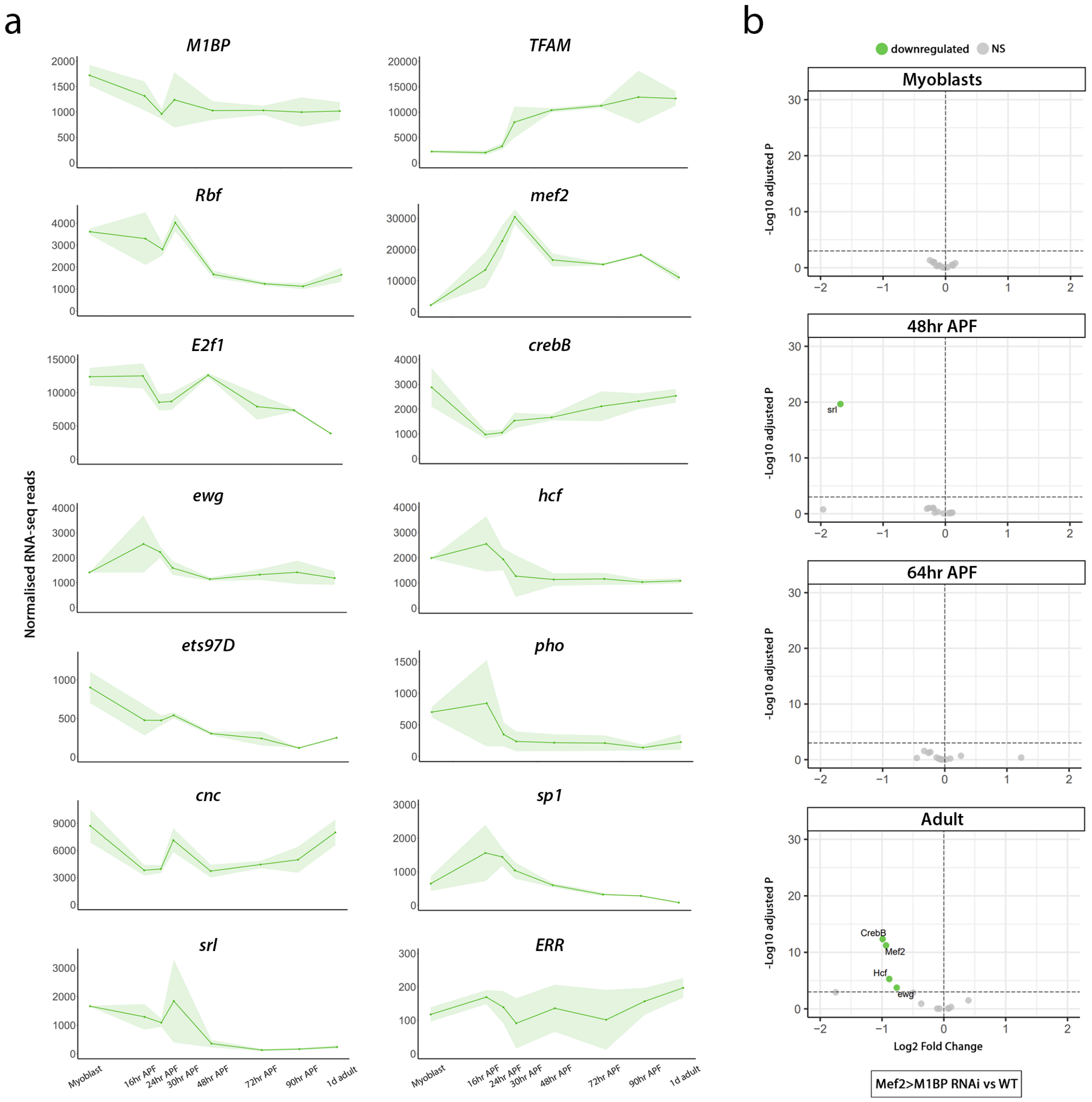

Supplementary Figure 7. M1BP does not transcriptionally regulate known OXPHOS activators

(a) Transcriptional profiles during flight muscle development of known OXPHOS transcriptional regulators. Mean and standard deviation are represented, data analysed from[7]. **(b)** Volcano plots of differential expression analysis of known OXPHOS transcriptional regulators between Mef2>WT and Mef>*M1BP* RNAi developing muscles. Significantly deregulated genes are depicted in green. For statistical analysis, the Wald test with Benjamini-Hochberg-correction was applied.

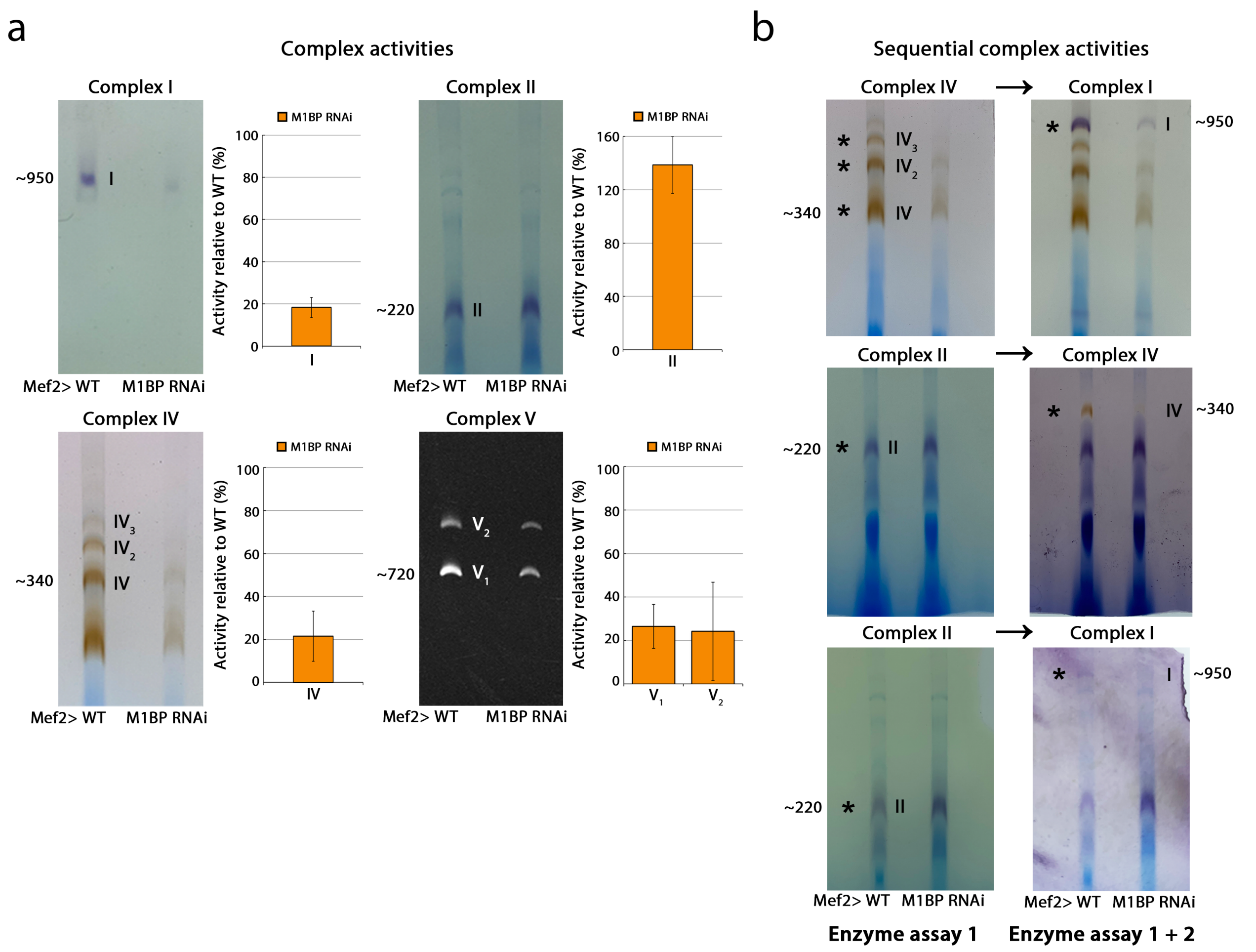

Supplementary Figure 8. OXPHOS complexes retain their enzymatic activities upon M1BP KD

(a) The activity of mitochondrial complexes upon M1BP KD was assayed following BN-PAGE separation of membrane-bound complexes. The intensity of the band representative of the complex activity after the incubation with a substrate was quantified for each complex. The intensity values are depicted as a percentage relative to the WT and represent a mean of two independent biological replicates. Approximate protein size is indicated in kDa. **(b)** The activity of mitochondrial complexes upon M1BP KD using *Mef2-GAL4* driver was assayed by BN-PAGE in a sequential manner, where the first tested activity is shown on the left, followed by the second activity test shown on the right. Approximate protein size is indicated in kDa. The experiment was performed in two independent biological replicates. Source data are provided as a Source Data File. Uncropped versions of the gels shown are provided at the end of the Supplementary Information.

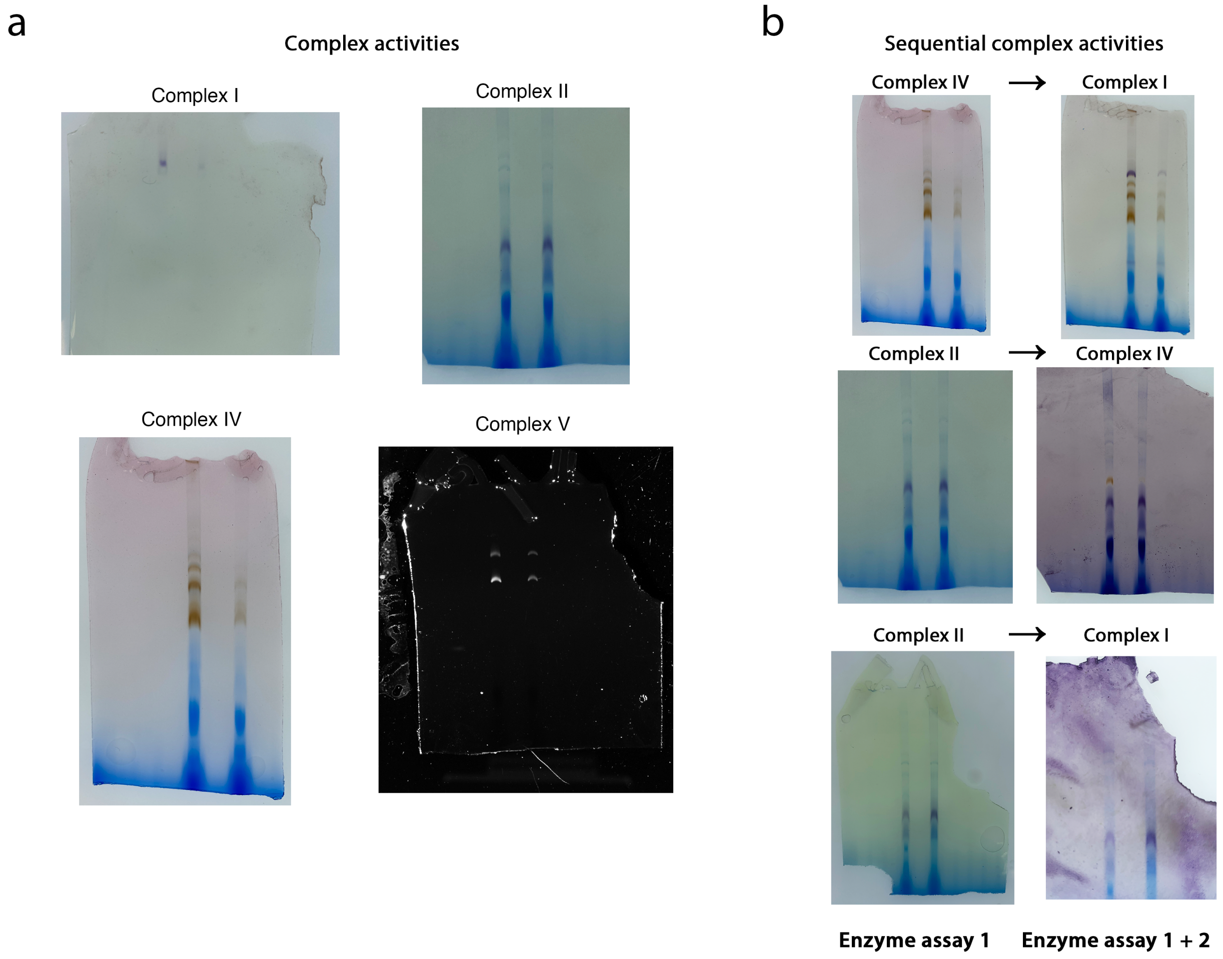

Source Data for Supplementary Figure 8.

The uncropped source data gels used in Supplementary Figure 8 are shown. The same layout as in Supplementary Figure 8 is used.
