# Supplementary material for: M1BP is an essential transcriptional activator of oxidative metabolism during Drosophila development
Source: Nat Commun. 2023 Jun 2;14:3187. doi: 10.1038/s41467-023-38986-5 (PMC10238438; doi:10.1038/s41467-023-38986-5)
Supplement: Supplementary file 3 — Description of Additional Supplementary Files [file 41467_2023_38986_MOESM3_ESM.pdf]

Description of Additional Supplementary Files

File Name: Supplementary Data 1
Description: Genes encoding proteins required for OXPHOS complex formation

File Name: Supplementary Data 2
Description: Differential gene expression analysis of M1BP RNAi in myoblasts

File Name: Supplementary Data 3
Description: Differential gene expression analysis of M1BP RNAi in 48 h APF DLMs

File Name: Supplementary Data 4
Description: Differential gene expression analysis of M1BP RNAi in 64 h APF DLMs

File Name: Supplementary Data 5
Description: Differential gene expression analysis of M1BP RNAi in adult DLMs

File Name: Supplementary Data 6
Description: Differential gene expression of mitochondrial DNA-encoded genes

File Name: Supplementary Data 7
Description: Identification of transcription factor binding sites in promoters of nuclear-encoded OXPHOS complex components

File Name: Supplementary Data 8
Description: Differential gene expression of genes involved in a cellular proteotoxic response

File Name: Supplementary Movie 1
Description: The inner mitochondrial membrane encompasses and isolates an aggregate. Tomographic reconstruction of a mitochondrial aggregate taken from dual-axis tomographic serial sections. Tomogram was joined and segmented with surface rendering showing the aggregate in gold and the encompassing inner mitochondrial membrane in cyan. Source data are provided as a Source Data File
