# Supplementary material for: M1BP is an essential transcriptional activator of oxidative metabolism during Drosophila development
Source: Nat Commun. 2023 Jun 2;14:3187. doi: 10.1038/s41467-023-38986-5 (PMC10238438; doi:10.1038/s41467-023-38986-5)
Supplement: Supplementary file 13 — Reporting Summary [file 41467_2023_38986_MOESM13_ESM.pdf]

nature portfolio

Corresponding author(s): Andrew Saurin

Last updated by author(s): 05/12/2023

Reporting Summary

Nature Portfolio wishes to improve the reproducibility of the work that we publish. This form provides structure for consistency and transparency in reporting. For further information on Nature Portfolio policies, see our Editorial Policies and the Editorial Policy Checklist.

Statistics

For all statistical analyses, confirm that the following items are present in the figure legend, table legend, main text, or Methods section.

n/a	Confirmed	
☐	☒	The exact sample size (*n*) for each experimental group/condition, given as a discrete number and unit of measurement
☐	☒	A statement on whether measurements were taken from distinct samples or whether the same sample was measured repeatedly
☐	☒	The statistical test(s) used AND whether they are one- or two-sided<br>*Only common tests should be described solely by name; describe more complex techniques in the Methods section.*
☒	☐	A description of all covariates tested
☐	☒	A description of any assumptions or corrections, such as tests of normality and adjustment for multiple comparisons
☐	☒	A full description of the statistical parameters including central tendency (e.g. means) or other basic estimates (e.g. regression coefficient) AND variation (e.g. standard deviation) or associated estimates of uncertainty (e.g. confidence intervals)
☐	☒	For null hypothesis testing, the test statistic (e.g. *F*, *t*, *r*) with confidence intervals, effect sizes, degrees of freedom and *P* value noted<br>*Give P values as exact values whenever suitable.*
☒	☐	For Bayesian analysis, information on the choice of priors and Markov chain Monte Carlo settings
☒	☐	For hierarchical and complex designs, identification of the appropriate level for tests and full reporting of outcomes
☒	☐	Estimates of effect sizes (e.g. Cohen's *d*, Pearson's *r*), indicating how they were calculated

Our web collection on statistics for biologists contains articles on many of the points above.

Software and code

Policy information about availability of computer code

Data collection	3DL_NuCount: deposited on Github. DOI 10.5281/zenodo.7695893 was used.
Data analysis	For data analysis the Fiji software PMID: 22743772 (version v1.53s) was used with following plugins : MyofibrilJ (PMID: 29846170), Cell counter (https://imagej.net/plugins/cell-counter) and Watershed (https://imagej.nih.gov/ij/plugins/watershed.html). For plot generation and analyses R software, version 4.1.0 (2021-05-18) was used. For high throughput sequencing analysis, DESeq2, version 1.34.0, was used. Tomographic reconstructions and tomograms joining were carried out in eTomo (IMOD, version 4.11), segmentation was carried out in Ilastik (version 1.4.0) and surface rendering in Amira (version 2022.2)

For manuscripts utilizing custom algorithms or software that are central to the research but not yet described in published literature, software must be made available to editors and reviewers. We strongly encourage code deposition in a community repository (e.g. GitHub). See the Nature Portfolio guidelines for submitting code & software for further information.

Data

Policy information about availability of data

All manuscripts must include a data availability statement. This statement should provide the following information, where applicable:

- Accession codes, unique identifiers, or web links for publicly available datasets
- A description of any restrictions on data availability
- For clinical datasets or third party data, please ensure that the statement adheres to our policy

All high throughput sequencing data have been deposited with the Gene Expression Ombudsman (GEO) and are available under the accession number GSE207241

Human research participants

Policy information about studies involving human research participants and Sex and Gender in Research.

Reporting on sex and gender	Not applicable
Population characteristics	Not applicable
Recruitment	Not applicable
Ethics oversight	Not applicable

Note that full information on the approval of the study protocol must also be provided in the manuscript.

Field-specific reporting

Please select the one below that is the best fit for your research. If you are not sure, read the appropriate sections before making your selection.

☒ Life sciences ☐ Behavioural & social sciences ☐ Ecological, evolutionary & environmental sciences

For a reference copy of the document with all sections, see nature.com/documents/nr-reporting-summary-flat.pdf

Life sciences study design

All studies must disclose on these points even when the disclosure is negative.

Sample size	For all imaging experiments, at least 8 animals with reproducible phenotype were analysed. For locomotory analyses, at least 50 animals with reproducible phenotype were analysed.
Data exclusions	No data exclusions were made
Replication	All experiments were repeated at least twice with highly similar results. Next generation sequencing was performed on three independent replicates
Randomization	All Drosophila were separated and grouped based on genetic background
Blinding	Experiment blinding was in most cases not possible, as is it the investigator that designed, performed the experiments and analysed the data, except for the flight test. In all experiments the same experimental conditions were applied to all samples to diminish bias.

Reporting for specific materials, systems and methods

We require information from authors about some types of materials, experimental systems and methods used in many studies. Here, indicate whether each material, system or method listed is relevant to your study. If you are not sure if a list item applies to your research, read the appropriate section before selecting a response.

Materials & experimental systems

n/a	Involved in the study
☐	☒ Antibodies
☒	☐ Eukaryotic cell lines
☒	☐ Palaeontology and archaeology
☐	☒ Animals and other organisms
☒	☐ Clinical data
☒	☐ Dual use research of concern

Methods

n/a	Involved in the study
☒	☐ ChIP-seq
☒	☐ Flow cytometry
☒	☐ MRI-based neuroimaging

Antibodies

Antibodies used

mouse ATP5A, 15H4C4 Thermo Scientific 43-9800, IF (1:200), WB (1:1000)
rabbit anti-M1BP (gift from David Gilmour), 1:250
mouse anti-Hsp70, StressMarq Biosciences Cat# SMC-106, RRID: AB_2295500, IF (1:100), WB (1:1000)
chicken anti-GFP, GFP-1010, AvesLabs (AB_2307313), 1:1000

AlexaFluor 488, goat anti-rabbit, Invitrogen, A11034, 1:500
AlexaFluor 568, goat anti-mouse, Invitrogen, A11004, 1:500
AlexaFluor 488, donkey anti-chicken A10039, Invitrogen, 1:500

Validation

anti-ATP5A antibody was validated in PMID:27529784 and PMID:25428350
anti-M1BP (D. Gilmour) antibody was validated in drosophila cell line in PMID: 28871058
mouse anti-Hsp70 antibody was validated for Immunufluorescence in PMID:29405094 and for Western Blot in PMID:26481195
For anti-GFP :
Antibodies were analyzed by western blot analysis (1:5000 dilution) and immunohistochemistry (1:500 dilution) using transgenic mice expressing the GFP gene product. Western blots were performed using BlokHen® (Aves Labs) as the blocking reagent, and HRP-labeled goat anti-chicken antibodies (Aves Labs, Cat. #H-1004) as the detection reagent. Immunohistochemistry used tetramethyl rhodamine-labeled anti-chicken IgY.

Animals and other research organisms

Policy information about studies involving animals; ARRIVE guidelines recommended for reporting animal research, and Sex and Gender in Research

Laboratory animals

Drosophila melanogaster strains as described in Methods and detailed here:
For M1BP downregulation the following lines were used: UAS-M1BP RNAi #1 (110498/KK VDRC) and UAS-M1BP RNAi #2 (BL32858). GAL4 drivers used in this study were muscle-specific Mef2-GAL4 (BL27390) and Him-GAL47, pan-neuronal elav-GAL4 line (BL458, C155) and fat body-specific cg-GAL4 line (BL7011). Myoblasts were visualised using a twi::GFP transgene (BL79615). Lines allowing mitochondria imaging in flight muscles were UAS-mit::Dendra239 and UAS-mit::mKate2 (chr. 2 and chr. 3) that was generated by P-element-mediated transgenesis using pUASp and contains the far-red fluorescent protein mKate2 (excitation 588nm, emission 633nm) fused at the N-terminus to human COXVIII mitochondrial target sequence (MSVLTPLLLRGLTGSARRLPV PRAKIHSL).

Wild animals

This study did not involve wild animals.

Reporting on sex

All experiments using adult animals were performed on females. The flight test was performed with males because of their greater performance in this assay. For experiments using larvae the sex was not discriminated.

Field-collected samples

This study did not involve field-collected samples.

Ethics oversight

This study on Drosophila melanogaster did not require ethical approval.

Note that full information on the approval of the study protocol must also be provided in the manuscript.
